# Ethnobotanical study of homegarden plants in Sebeta-Awas District of the Oromia Region of Ethiopia to assess use, species diversity and management practices

**DOI:** 10.1186/s13002-015-0049-8

**Published:** 2015-08-22

**Authors:** Tefera Mekonen, Mirutse Giday, Ensermu Kelbessa

**Affiliations:** Environmental Science Program, Addis Ababa University, P. O. Box 1176, Addis Ababa, Ethiopia; Aklilu Lemma Institute of Pathobiology, Addis Ababa University, P. O. Box 1176, Addis Ababa, Ethiopia; The National Herbarium, Addis Ababa University, P. O. Box 1176, Addis Ababa, Ethiopia

**Keywords:** Homegarden, Agrobiodiversity, Local knowledge, Sebeta-Awas, Ethiopia

## Abstract

**Background:**

Homegardens in Ethiopia are currently facing different threats mainly due genetic erosion, loss of traditional knowledge on their use and management and drought. On the other hand, research and documentation works on homegardens in the country are very limited. There is no previous report indicating conduct of ethnobotanical study on homegardens in selected study district. The present study thus attempted to document knowledge on uses and management practices of homegardens by people in study district.

**Methods:**

The study was conducted in Sebeta-Awas District, Southwestern Shewa Zone of Oromia Region, Ethiopia, between March and September 2009 to assess use, species diversity and conservation status of homegardens in the District. Data were collected using semi-structured interviews as well as through homegarden visits, market surveys and different ranking exercises. For the semi-structured interviews, 42 homegarden owners were selected randomly from seven sampled kebeles (smallest administrative units in Ethiopia), six from each kebele. For different ranking exercises, 14 informants (10 males and 4 females) were sampled using convenient sampling method from among homegarden owners that already participated in semi-structured interviews.

**Results:**

In total, 113 plant species belonging to 46 families were recorded from the study area, of which 45 (39.8 %) were herbs, 34 (30.1 %) were trees, 26 (23.0 %) were shrubs and 8 (7.1 %) were climbers. Fabaceae had the highest number of species, followed by the families Asteraceae, Lamiaceae and Solanaceae. The cash crops *Catha edulis, Rhamnus prinoides* and *Ruta chalepensis* were the most frequently encountered homegarden plants. *Cupressus lusitanica*, *Eucalyptus camaldulensis* and *Faidherbia albida* were the most abundant tree species that had the highest densities of occurrence. Of the recorded plant species, 25 % were used as sources of food, 13 % as medicine and 10 % as household tools.

**Conclusion:**

It can be concluded that homegardens in the study area are rich in crops and, therefore, significantly contribute to the agrobiodiversity of the study District, in particular, and Ethiopia, in general.

## Background

Homegarden is commonly defined as a piece of land with a definite boundary surrounding a homestead, being cultivated with diverse mixture of perennial and annual plant species, arranged in a multilayered vertical structure, often in combination with raising livestock, and managed mainly by household members for subsistence production [1–4]. Homegardens are complex ecosystems close to the house where plants can be closely observed and managed and are convenient place for traditional plant experimentation [5].

Homegardens are important in the conservation of useful plant species since they contain very large numbers of species which are often absent or disappearing from other production systems [6]. Homegardens also provide a wide range of ecological benefits and services and a valuable set of products for the rural poor [6]. Homegardens provide people with supplementary food, fuel and fodder [7]. They are used to grow medicinal, spice, ornamental and stimulant plants [8]. Homegardens are widely spread in the tropical and subtropical regions of Asia [9], Africa [10] and Central and South America [11].

Although there is no direct evidence as to when homegardening started in Ethiopia, based on the antiquity of agriculture, crop composition, oral literature and rich vernacular designations in different local languages, it is assumed to have long history [12]. Homegardening in Ethiopia is estimated to have started as early as 5000 to 7000 years ago, around the time when agriculture started in the country [13, 14]. In relation to the house, Ethiopian homegardens may occupy different positions such as the backyards, frontyards, side yards and yards that almost encircle the house, and have variable shapes and sizes and composition of plant species [12]. In Ethiopia, homegardens are prevalent in the highland areas and mainly accommodate supplementary fruits and vegetables as a principal means of livelihood for households [15–18]. A study [16] indicated that more than 170 crop species belonging to 121 genera and 50 families have been recorded in Ethiopia, of which, the families Fabaceae, Lamiaceae, Poaceae, Rubiaceae, Asteraceae, and Rubiaceae contributed more than 10 species each.

Ethiopia is one of the eight world centers of origin and diversity of agricultural products [19] which is partly the result of *in situ* conservation of plants traditionally grown in homegardens [12, 17]. However, homegardens are currently threatened mainly due to genetic erosion, loss of traditional knowledge of different management practices, man-made habitat changes, and drought [4, 20]. On the other hand, research and documentation works done on homegardens in Ethiopia are very limited [21–26] and most of them have been conducted in the south and southwestern parts of the country. There is no report indicating the conduct of ethnobotanical study on homegardens of the selected district. The present study thus attempted to gather and document information on the use of plant species and management practices of homegardens by people in Sebeta-Awas District, Southwestern Shewa Zone of Oromia Region, Ethiopia.

## Methods

### The study area

**Fig. 1:**
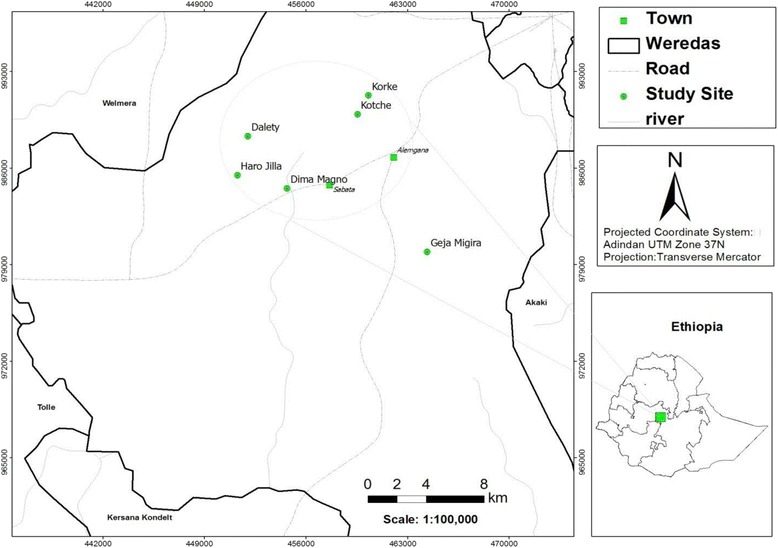
Map of Sebeta-Awas District locating the selected study kebeles (drawn based on Ethio GIS Data)

This study was conducted in Sebeta-Awas District (Fig. 1), Oromia Region, Ethiopia, which is located at a distance of 24 km to 45 km southwest of the capital Addis Ababa. The District has an area of 87,532 ha. It shares borders with Akaki District in the East, Kerssa and Tole districts in the south, Welmera District in the North and Ilu and Ejere districts in the West. The land feature of Sebeta-Awas is characterized by mountains and hills (Wachacha and Hoche mountains) and marshy plains (Furi-Gara-Bello, Gejja Ballachis and Jammo), and is surrounded by Awash water shade in the west [27]. Altitude in the District ranges between 1800 and 3385 m a.s.l (Sabata Awas District Rural and Agricultural Office, unpublished data of 2001).

Agricultural activity is the dominant means of livelihood in Sebeta-Awas District. People in the District use functional categorization to classify their lands, e.g., grazing land, agricultural land, homestead land and forestland. According to annual report of Sebeta-Awas District, Rural and Agricultural Development Office, out of 87,532 ha of land, 73,838 ha (84.4 %) are used for agriculture to cultivate different crops for household consumption and sale in local market, and 3.689 ha (4.2 %) of land is used as grazing area (Sabata Awas District Rural and Agricultural Office, unpublished data of 2006).

The District is divided into two agro-ecological zones locally called *Baddaa* (12 %) and *Badda daree* (88 %), which means highland and midland areas, respectively (Sabata Awas District Rural and Agricultural Office, unpublished data of 2006). The study area experiences alternating wet and dry seasons. The main rainy season is between June and September and is locally called *rooba gaanaa*. Light rain occurs between January and March and is locally called *rooba arfassa*. There is no temperature data for Sebeta-Awas District. Thus temperature data of Alemtena, a nearby district having similar altitude as that of Sebeta-Awas District, was used to compute 10 years (1995 to 2006) annual mean temperature for the study area. The annual mean maximum and minimum temperatures are 25.4 °C and 13.9 °C, respectively. The annual mean maximum and the minimum temperatures were recorded in the months of May and July, respectively. The total mean annual rainfall from 1995 to 2006 is 1054.7 mm and the highest rainfall recorded is for July (National Metrological Service of Ethiopia, unpublished data of 2009).

### Selection of study kebeles and homegardens

Reconnaissance survey was conducted in Sebeta-Awas District in January 2009 to select study kebeles (smallest administrative units in Ethiopia) and conduct homegarden surveys. During the reconnaissance survey, seven kebeles (from a total of 42 kebeles in the District) including Dima Guranda, Dalety, Dima Magno, Geja Migra, Haro Jilla, Korke and Kotche were randomly selected. In each selected kebele, visits were made to 50 households that were sampled during random walks to check for the presence or absence of homegardens and assess their sizes, shapes and locations with respect to houses.

### Selection of informants and ethnobotanical data collection

Ethnobotanical data were collected between March and August 2009 mainly through homegarden tours, market surveys and semi-structured interviews. Interviews were conducted using pre-prepared questions with 42 randomly selected homegarden owners (32 males and 10 females with ages ranging from 35 to 72 years) from the randomly selected seven kebeles, six from each kebele, after receiving their full consent. The homegarden owners involved in the interviews were sampled from the list of households that were found to own homegardens during survey of 350 households (50 from each sampled kebele) to check for the presence or absence of homegardens. All interviews were conducted in Oromo language. During interviews with the informants, attempts were made to document information on the use of homegarden plants for different purposes (household food supply, medicine, shade, aesthetics and ornament, fuel wood production and for income generation) and management practices. Additional data were also gathered through, simple preference ranking, direct matrix ranking and paired comparison exercises [28–30] by involving 14 informants (two from each kebele) between the ages of 40 and 65 (10 males and 4 females). The informants were sampled using convenient sampling method from among homegarden owners that already participated in the semi-structured interviews. The study was conducted in accordance with the Code of Ethics of the International Society of Ethnobiology [31].

#### Simple preference ranking

During preference ranking exercise, 14 informants were asked to rank seven marketable homegarden plants in Sebeta-Awas District that were found to have the highest frequencies and relative frequencies of occurrence and rank them according to their perceived values or desirability following the method of Martin [30]. The integer values 1, 2, 3 and 4 were used whereby the most important plant was given the highest value, while the least important one was assigned with the smallest value. The numbers were then summed for all respondents to come up with an overall ranking.

#### Direct matrix ranking

Direct matrix ranking exercises were conducted by the same 14 informants on six multipurpose tree species as reported during interviews using the approach of Martin [30] to rank them based on their uses as construction material, fertilizer, household tools, charcoal/firewood, shade, live fence and medicine. Informants were asked to assign value to each attribute (5 = best, 4 = very good, 3 = good, 2 = less used, 1 = least used and 0 = not used).

#### Paired comparison

Paired comparison exercises were conducted by the 14 informants following the methods of Martin [30] on five nutraceutical plants (plants used as sources of both food and medicine) as reported during interviews with informants.

#### Market survey

Market survey was conducted in one open market found in Sebeta, an administrative town of the Sebeta-Awas District, to check for availability of marketable homegarden plants. Data were gathered through observation and interaction with sellers and buyers of homegarden products.

### Plant specimen collections and identification

Specimens of plants recorded as homegarden species were collected, numbered, pressed and dried. They were later identified using taxonomic keys of the Flora of Ethiopia and Eritrea and by comparison with already identified specimens that were deposited at National Herbarium (ETH), Addis Ababa University. The identities of the specimens were authenticated by taxonomists at ETH.

### Data analysis

Descriptive statistical methods were employed to determine frequencies, relative frequencies, densities and relative densities. Shannon and Wiener index and Sorensen’s Index were used to estimate species diversity and similarity, respectively, in sample plots of 15 m x 15 m (225 m^2^) in homegardens of the 42 randomly selected informants, six from each of the seven selected kebeles.

#### Frequency and relative frequency

Frequencies and relative frequencies were calculated for plants in the sampled homegardens. Frequency describes the distribution of a species throughout the stands. It is determined by calculating the percentage of plots/quadrants in a sample area on which a given species occurs [30].

$$ \mathrm{Frequency}=\frac{\mathrm{quadrants}\ \mathrm{in}\ \mathrm{which}\ \mathrm{a}\ \mathrm{species}\ \mathrm{occurs}}{\mathrm{Total}\ \mathrm{number}\ \mathrm{of}\ \mathrm{quadrants}\ \mathrm{in}\ \mathrm{the}\ \mathrm{sample}}\times 100 $$

Relative frequency is the number of occurrences of a species as a percentage of the total occurrences of all species [30].

$$ \mathrm{Relative}\ \mathrm{frequency}=\frac{\mathrm{Frequency}\ \mathrm{of}\ \mathrm{a}\ \mathrm{species}\ \mathrm{in}\ \mathrm{the}\ \mathrm{sample}}{\mathrm{Total}\ \mathrm{frequency}\ \mathrm{of}\ \mathrm{a}\mathrm{ll}\ \mathrm{species}\ \mathrm{in}\ \mathrm{the}\ \mathrm{sample}}\times 100 $$

#### Density and relative density

Density is the average number of individuals of a species on a unit area basis. It is closely related to abundance but more useful in estimating the importance of a species [30].

$$ \mathrm{Density}=\frac{\mathrm{Number}\ \mathrm{of}\ \mathrm{in}\mathrm{dividuals}\ \mathrm{in}\ \mathrm{the}\ \mathrm{sample}}{\mathrm{Total}\ \mathrm{area}\ \mathrm{of}\ \mathrm{the}\ \mathrm{sample}\ \left({\mathrm{m}}^2\right)} $$

Relative density is the number of individuals of a species as a percentage of the total number of individuals of all species in that area [30].

$$ \mathrm{Relative}\ \mathrm{density}=\frac{\mathrm{Number}\ \mathrm{of}\ \mathrm{in}\mathrm{dividuals}\ \mathrm{of}\ \mathrm{a}\ \mathrm{species}\ \mathrm{in}\ \mathrm{the}\ \mathrm{sample}}{\mathrm{Total}\ \mathrm{number}\ \mathrm{of}\ \mathrm{in}\mathrm{dividuals}\ \mathrm{of}\ \mathrm{a}\mathrm{ll}\ \mathrm{species}\ \mathrm{in}\ \mathrm{the}\ \mathrm{sample}}\times 100 $$

Multipurpose trees species occurring in home gardens of the study area were considered in the computation of densities and relative densities.

#### Similarity and diversity indices

Sorenson’s Index of Similarity was used to compare the degree of similarity of species in the 42 homegardens randomly selected from the seven study kebeles (6 homegardens in each kebele) based on the species composition of quadrats [32]. It was calculated using the formula Ss = 2a/2a + b + c, where S_S_ **=** Sorensen’s similarity coefficient, a = number of species common to quadrat, b = number of species in quadrat 1 and c = number of species in quadrats 2. The coefficient values range from 0 (complete dissimilarity) to 1 (total similarity). This method was applied in all the 42 homegardens in the selected kebeles.

The Shannon-Weiner Index [33] was used to calculate and compare species diversity in the 42 homegardens in the seven study kebeles. It was calculated using the formula H = ∑ − (P_i_ ln P_i_), where H = the Shannon diversity index, P_i_ = fraction of the entire population made up of species I, S = numbers of species encountered, ∑ = sum from species 1 to species S and ‘ln’ is the natural logarithm to the base e (log _e_).

## Results and discussion

### Distribution, location and plant composition of homegardens

Out of the 350 houses surveyed in the seven selected kebeles in the study District, 248 (70.9 %) had homegardens, of which 126 (36 %), were located in the backyard (Table 1). Homegardens had different sizes and shapes, and served as animal houses, grain stores and land for growing different plant species. The size of homegardens sampled ranged from 300 m^2^ to 1200 m^2^. Distinct variation in size, diversity and composition of species was observed among homegardens. With increasing size of homegardens, more richness of species composition was observed. A similar study conducted in southern Ethiopia [24] revealed that as the size of homegarden increases, so does the diversity of plant species. Concerning distance, some homegardens were located very close to houses, where as others were found at places a bit far from houses (at a walking distance of 5–7 min).

**Table 1:** Distribution and location of homegardens in the selected seven study kebeles of Sebeta-Awas District

Kebeles	No. of surveyed houses	No. of houses with homegardens	Position of homegardens						
			front yard gardens	backyard gardens	side yard gardens	front yard & backyard gardens	front yard & side yard gardens	backyard & side yard gardens	round yard gardens
Dima Guranda	50	39	5	12	-	3	-	4	1 5
Dima Magno	50	35	-	18	5		-	-	12
Haro Jila	50	38		22	4	10			2
Dalati	50	34	6	18	2	-	7	1	-
Geja Migra	50	33	4	14	-	3	3	8	1
Koche	50	37	14	22	-	-	-	-	1
Korke	50	32	-	20	-	7	-	5	-
Total	350	248	29	126	11	23	10	18	31
%		70.9	8.3	36	3.1	6.6	2.9	5.1	8.9

Homegarden plants in the study area were composed of trees, shrubs, herbs and climbers in different strata. They consisted of trees approximately 10 to 15 m tall on the upper strata (e.g., *Cordia Africana*), fruit crops 1 to 10 m tall in the middle strata *(*e.g., *Citrus sinensis)* and herbaceous plants up to 1 m tall on the ground strata (e.g., *Brassica carinata* and *Cymbopogon citratus*).

### Management of homegardens

People in the study area have developed homegardens (locally called *eddo*) with considerable diversity and flexibility that support production of major livelihood crops. They have managed to select crops that co-adapt the local environment and that give multiple benefits. Some homegarden owners reported that they grew vegetables during the rainy season as well as the dry season by fetching water from where it is available. Some homegarden owners stated that they continuously manage plants for economic as well as ecological benefits. Crop residues, weeds, ashes and manures were reported to be used as fertilizers in homegardens. Few homegarden owners reported efforts made to reduce soil depletion in erosion-prone areas by planting shrubs (e.g., *Rosmarinus officinalis*) near the homestead.

Homegardens in the study area were open plots, or fenced or semi-fenced areas. Trees and shrubs were used as live fences to protect homegarden plants from predators. Management of homegardens was done through division of labor among family members. Observation and conversation with informants revealed that females played more roles than males in managing homegardens. Females were more involved in weeding, watering and planting, whereas, males’ activity was limited to fencing. Dominance of females in hoeing, weeding, and harvesting has been indicated in works conducted in Tanzania [34] and Ghana [35]. Selection of crops or vegetables for homegardening has been done in consultation with household members although the final decision was left to women. Despite the fact that management of homegardens in the study area was mainly the responsibility of females, as explained by women homegarden owners, access to or control over its benefits depends on the type of production. Men had more control on major income crops (e.g., *Chata edulis*). Minor income generated from crops such as vegetables is controlled by women. In homegardens dominated by subsistence crops, females did most of the work. However, in homegardens dominated by cash crop fruit trees, women’s participation was very minimal. Such a clear gender division in homegarden responsibilities is frequently recorded in the literature, e.g., Vietnam [36] and Mexico [37] and Peru [38].

Exchange of seeds of selected varieties and knowledge among homegarden owners was reported to be common practices in the study area. Exchange of information regarding homegardens among relatives, friends, and neighbors played a role in maintaining local cultural knowledge and practices in the study area. Exchange of plant resources and information among local communities is essential for agrobiodiversity conservation [30].

### Richness and diversity of homegarden plant species

Out of 350 houses visited in the seven selected kebeles, 248 (70.9 %) had homegardens, of which 42 (6 from each kebele) were selected for detailed interview surveys. The interview survey revealed 113 homegarden plant species belonging to 46 families (Table 2) which supports the assertion that homegardens are valuable sources of plant agrobiodiversity [39]. It was found out that the family Fabaceae had the highest number of species (15 spp.), followed by Asteraceae (10 spp.), Lamiaceae (7 spp.), Solanaceae (6 spp.), Poaceae and Rosaceae (5 spp. each), and Myrtaceae and Rutaceae (4 species each). Five families had three species each. Other nine families had 2 species each and 24 families had one species each. A study conducted in Loma and Gena Bosa districts of Ethiopia also reported the high number of homegarden plants belonging to the families of Fabaceae, Asteraceae and Poaceae [8]. Of the total species, 45 (39.8 %), were herbs, 34 (30.1 %) were trees, 26 (23.0 %) were shrubs and 8 (7.1 %) were climbers. *Cupressus lusitanica, Eucalyptus camaldulensis Eucalyptus globules* and *Grevellea robusta* were the canopy tree species. Among shrub species, *Catha edulis, Rosmarinus officinalis* and *Rhamnus prinoides* were the most prominent ones.

**Table 2:** Homegarden plants documented from Sebeta-Awas District

Family	Scientific name	Local name	Habit	Indigenous/exotic	Use	Coll. No.
Acanthaceae	*Justicia schimperiana* (Hochst. ex Nees) T.Anders.	Dhummugaa (O)	Shrub	Indigenous	Live fence	TM75
Alliaceae	*Allium cepa* L.	Qullubii-diimaa (O)	Herb	Exotic	Vegetable	TM105
	*Allium sativum* L.	Quulubii-adii (O)	Herb	Exotic	Vegetable, medicine	TM76
Amaranthaceae	*Amaranthus hybridus* L.	Oromee (O)	Herb	Exotic	Vegetable, weed	TM32
	*Achyranthes aspera* L.	-	Herb	Indigenous	Medicine, weed	TM92
	*Iresine herbstii* Hook. ex. Lindl.	-	Herb	Exotic	Ornament	TM16
Anacardiaceae	*Rhus glutinosa* A. Rich	Xaxeecha (O)	Tree	Indigenous	Fuel wood	TM104
	*Schinus molle* L.	Alaaltu (O)	Tree	Exotic	Shade, household tool	TM63
	*Mangifera indica* L.	Mango (O, A, G)	Tree	Exotic	Fruit crop	TM45
Annonaceae	*Annona cherimola* Mill.	Gishta (A)	Tree	Exotic	Fruit crop	TM87
Apiaceae	*Anethum graveolens* L.	-	Herb	Exotic	Weed	TM10
	*Daucus carota* L.	Carorot (A)	Herb	Indigenous	Vegetable	TM57
Apocynaceae	*Carissa spinarum* L.	Hagamsa (O)	Liana	Indigenous	Live fence	TM70
Asparagaceae	*Agave americana* L.	Argissa (O)	Herb	Exotic	Household tool	TM47
	*Agave sisalana* Perrine ex. Engler	Argissa (O)	Herb	Exotic	Household tool	TM79
Asteraceae	*Artemisia absinthum* L.	Arrity	Herb	Exotic	Medicine	TM24
	*Conyza pyrrhopappa* Sch. Bip ex A. Rich.	-	Herb	Indigenous	Ornament	TM109
	*Guizotia schimperi* Sch.Bip ex. Walp	-	Herb	Indigenous	Weed	TM39
	*Lactuca sativa* L.	Selata (A)	Herb	Exotic	Vegetable	TM108
	*Parthenium hysterophorous* L.	Faramsiisa (O)	Herb	Exotic	Weed	TM107
	*Silybum marianum* (L.) Gaertn.	Sokooruu (O)	Herb	Exotic	Weed	TM99
	*Sonchus oleraceus* L.	Sokooruu (O)	Herb	Exotic	Weed	TM58
	*Spathodea campanulata* P. Beauv.	-	Tree	Exotic	Shade	TM65
	*Tagetes patula* L.	-	Herb	Exotic	Ornament	TM89
	*Vernonia amygdalina* Del.	Ebichaa (O)	Shrub	Indigenous	Household tool, medicine	TM19
Boraginaceae	*Cordia africana* Lam.	Wadeecha (O	Tree	Indigenous	Household tool, shade, medicine	TM15
Brassicaceae	*Brassica carinata* A. Br.	Yeguragiegomen (A)	Herb	Indigenous	Vegetable	TM111
	*Brassica oleracea* L.	Goommana (O), Tql-gomen (A)	Herb	Exotic	Vegetable	TM93, TM101
Cactaceae	*Opuntia cylindrica* (Lam.) DC.	Qulqal (A)	Shrub	Exotic	Live fence	TM30
Caricaceae	*Carica papaya* L.	Papay (A)	Tree	Exotic	Fruit crop, medicine	TM7
Celastraceae	*Catha edulis* (Vahl) Forssk. ex Endl.	Caatii (O)	Shrub	Indigenous	Stimulant	TM6
Chenopodiaceae	*Beta vulgaris* L.	Qosta (A)	Herb	Exotic	Vegetable	TM20
Cucurbitaceae	*Cucurbita pepo* L.	Dabaaqula (O)	Liana	Exotic	Vegetable, medicine	TM46
	*Lagenaria abyssinica* (Hook. f.) C. Jeffery	Hadoftu (O)	Liana	Indigenous	Household tool	TM59
Cupresaceae	*Cupressus lusitanica* Mill.	Gaattiraa–faraanjii (O)	Tree	Exotic	Live fence	TM48
	*Juniperus procera* Hochst. ex Endl.	Gatira Habasha (O)	Tree	Indigenous	Construction, household tool	TM44
Cyperaceae	*Cyperus rotundus* L.	-	Herb	Indigenous	Household tool	TM13
Euphorbiacaeae	*Croton macrostachyus* Del.	Bakkanisa (O)	Tree	Indigenous	Fuel wood, shade, household tool	TM60
	*Euphorbia ampliphylla* Pax.	Adamee (O)	Herb	Indigenous	Weed	TM36
	*Ricinus communis* L.	Qobboo (O)	Herb	Indigenous	Household tool	TM78
Fabaceae	*Acacia abyssinica* Hochst. ex Benth.	Laaftto (O)	Tree	Indigenous	Fuel wood, shade, household tool	TM9
	*Faidherbia albida* (Delile) A. Chev.	Laftto (O)	Tree	Indigenous	Shade, household tool	TM1
	*Acacia mearnsii* De Willd.	Yetemenja–zaf (O)	Tree	Exotic	Fuel wood	TM18
	*Acacia saligna* (Labill.) Wendl.	-	Tree	Exotic	Shade	TM4
	*Albizia schimperiana* Oliv.	Hambabessa (O)	Tree	Indigenous	Shade, household tool	TM8
	*Caesalpinia decapetala* (Roth) Alston	Arangamaa (O)	Liana	Exotic	Live fence	TM3
	*Calpurnia aurea* (Ait.) Benth.	Ceekaa (O)	Shrub	Indigenous	Medicine	TM68
	*Erythrina brucei* Schweinf.	Wolensuu (O)	Tree	Indigenous	Shade	TM52
	*Millettia ferruginea* (Hochst.) Bak.	Sotoollo (O)	Tree	Indigenous	Shade, household tool	TM77
	*Phaseolus lunatus* L.	Adengware (A)	Liana	Exotic	Pulse	TM49
	*Phaseolus vulgaris* L.	Boloqqie (A)	Liana	Exotic	Pulse	TM71
	*Senna septemtrionalis* (Viv.) Irwin & Barnby	Akayi-warabessa (O)	Tree	Exotic	Fuel wood	TM17
	*Sesbania sesban* (L.) Merr.	-	Shrub	Exotic	Live fence	TM35
	*Trifolium tembense* Fresen.	-	Herb	Indigenous	Weed	TM112
	*Vicia faba* L.	Baqeella (O)	Herb	Exotic	Pulse	TM110
Flacourtiaceae	*Dovyalis caffra* (Hook. f. & Harv.) Hook. f.	Koshomii (O)	Shrub	Exotic	Live fence	TM80
Lamiaceae	*Mentha longifolia* (L.) Hudson	-	Herb	Indigenous	fragrant	TM40
	*Mentha puegium* L.	Nana (A)	Herb	Indigenous	Spice	TM94
	*Ocimum basilicum* L.	Besobilla (A)	Herb	Exotic	Spice	TM74
	*Ocimum lamiifolium* Hochst. ex Benth.	Koricha–Michii (O)	Shrub	Indigenous	Medicine	TM66
	*Ocimum urticifolium* Roth	Koricha-alkani (O)	Shrub	Indigenous	Medicine	TM100
	*Otostegia integrifolia* Benth.	Tungit (A)	Shrub	Indigenous	Medicine	TM106
	*Rosmarinus officinalis* L.	Siga-metibesha (A)	Shrub	Exotic	Fragrant	TM31
Lauraceae	*Persea americana* Mill.	Abukado (O, A, G)	Tree	Exotic	Fruit crop	TM2
Loganiaceae	*Buddleja davidii* Franch.	Amfar (O)	Shrub	Exotic	Live fence	TM82
Lythraceaee	*Punica granatum* L.	Roman (A)	Shrub	Exotic	Fruit crop, medicine	TM85
Malvaceae	*Hibiscus* sp.	-	Shrub	Indigenous	Ornament	TM25
	*Malva verticillata* L.	Litii (O)	Herb	Indigenous	Medicine	TM64
	*Sida schimperiana* Hochst. ex. A. Rich.	Guute (O)	Herb	Indigenous	Household tool	TM103
Moraceae	*Ficus elastica* Roxb.	Yegoma-zaf (A)	Tree	Exotic	Shade	TM95
	*Ficus sur* Forssk.	Harbuu (O)	Tree	Indigenous	Shade	TM29
	*Morus alba* L.	Enjorie (O)	Tree	Exotic	Fruit crop	TM28
Musaceae	*Ensete ventricosum* (Welw.) Cheesman.	Qoccoo (O)	Herb	Indigenous	Medicine, household tool	TM41
Myrtaceae	*Eucalyptus camaldulensis* Dehnh.	Bargamo-diimaa (O)	Tree	Exotic	Construction, fuel wood, live fence	TM5
	*Eucalyptus globulus* Labill.	Bargamo-adii (O)	Tree	Exotic	Construction, medicine	TM27
	*Myrtus communis* L.	Ades (A, G)	Herb	Exotic	Fragrant	TM86
	*Psidium guajava* L.	Zeytunaa (O)	Tree	Exotic	Fruit crop	TM53
Nyctaginaceae	*Bougainvillea glabra* Choisy	Bugambe (A)	Liana	Exotic	Ornament	TM51
Oleaceae	*Jasminum abyssinicum* Hochest. ex DC.	Qamaxxee (O)	Liana	Indigenous	Medicine	TM91
	*Olea europaea* L. subsp. *cuspidat*a (Wall. ex G. Don) Cif.	Ejeersa (O)	Tree	Indigenous	Fragrant	TM22
Phytolaccaceae	*Phytolacca dodecandra* L’ Herit.	Endod (A)	Shrub	Indigenous	Household tool	TM81
Pinaceae	*Pinus patula* Schiede ex. Schltdl. Cham.	Arzelibanose (A)	Tree	Exotic	Live fence	TM33
Poaceae	*Arundo donax* L.	Shambako (O)	Herb	Exotic	Household tool, live fence	TM97
	*Cynodon dactylon* (L.) Pers.	Coqoorsa (O)	Herb	Indigenous	Household tool	TM90
	*Cymbopogon citratus* (DC.) Stapf	Tej-sar (A)	Herb	Exotic	Fragrant, medicine	TM21
	*Saccharum officinarum* L.	Shenkor-ageda (A)	Herb	Exotic	Sugar crop	TM54
	*Zea mays* L.	Boqqolloo (O)	Herb	Exotic	Cereal crop	TM113
Poygonaceae	*Rumex nepalensis* Spreng.	Shultii (O)	Herb	Indigenous	Medicine	TM88
Proteaceae	*Grevillea robusta* R. Br.	-	Tree	Exotic	Live fence, shade	TM50
Rhamnaceae	*Rhamnus prinoides* L’ Herit.	Geeeshoo (O, A)	Shrub	Indigenous	Household tool	TM14
Rosaceae	*Hagenia abyssinica* (Bruce) J. F. Gmel.	Heexoo (O)	Tree	Indigenous	Medicine	TM61
	*Malus sylvestris* Miller	Apple	Tree	Exotic	Fruit crop	TM98
	*Prunus x domestica* L.	Prim (A)	Tree	Exotic	Fruit crop	TM69
	*Rosa abyssinica* Lindley	Qega (A)	Shrub	Indigenous	Live fence	TM42
	*Rosa hybrida* L.	Tsigereda (A)	Shrub	Exotic	Ornament	TM12
Rubiaceae	*Coffea arabica* L.	Buna (O, A, G)	Shrub	Indigenous	Stimulant	TM11
	*Galium aparinoides* Forssk.	Maxaanee (O)	Herb	Indigenous	Weed	TM114
Rutaceae	*Casimiroa edulis* La Llave	Shasho (A)	Tree	Exotic	Fruit crop	TM43
	*Citrus aurantifolia* (Christm.) Swingle	Lomii (O)	Shrub	Exotic	Fruit crop	TM37
	*Citrus sinensis* (L.) Osb.	Burtukaana (O)	Shrub	Exotic	Fruit crop	TM83
	*Ruta chalepensis* L.	Chiracot (O, G)	Herb	Exotic	Spice, medicine	TM67
Salicaceae	*Populus* sp.	Ye-kibrit enchet (A)	Tree	Exotic	Household tool	TM23
Santalaceae	*Osyris quandripartita* Decn.	Watoo (O)	Tree	Indigenous	Construction	TM62
Scrophulariaceae	*Verbascum sinaiticum* Benth.	Guraa haree	Shrub	Indigenous	Weed	TM56
Simarobaceae	*Brucea antidysenterica* J. F Mill.	Qomeenyo (O)	Shrub	Indigenous	Medicine	TM38
Solanaceae	*Capsicum annuum* L.	Brbare (O)	Herb	Exotic	Vegetable	TM34
	*Datura stramonium* L.	Asangiraa (O)	Herb	Indigenous	Medicine	TM102
	*Lycopersicon esculentum* Mill.	Timatimi (O)	Herb	Exotic	Vegetable	TM73
	*Nicotiana tabacum* L.	Tamboo (O)	Herb	Exotic	Stimulant	TM55
	*Solanum tuberosum* L.	Dinichaa (O)	Herb	Exotic	Tuber crop	TM84
	*Withania somnifera* (L.) Dunal	Gizawa (A)	Herb	Indigenous	Medicine	TM72
Verbenaceae	*Lantana camara* L.	Ye- wof kolo (A)	Shrub	Exotic	Ornament	TM26
	*Lippia abyssinica* (Otto & A. Dietr.) Cufod.	Kusaaye (O)	Shrub	Indigenous	Spice, fragrant	TM96

*A* Amharic Language, *O* Oromo language

Of the total reported homegarden plants, 63 (58 %) were found to be exotic species and 51 (48 %) were indigenous species (Table 2). The fact that the majority of homegarden plants were exotic might be attributed to their management suitability. Of the exotic plants, relatively higher number of species (24) was used as food plants and eight species were used as live fence. Relatively higher number of the indigenous plants (13 species) was used as source of medicine and nine species were used as household tools.

Of the study kebeles, Dima Guranda had the highest species diversity (H’ = 3.555), followed by Haro Jilla (H’ = 3.497). The lowest diversity index (H’ = 3.348) was computed for Dalety (H’ = 2.890) (Table 3). Homegarden owners reported that homegarden species diversity could be related to factors such as access to water, size of homegarden and infestation of plants by pests and weeds. Elsewhere, it was reported that low species diversity could be a result of the shifting from polycultural gardening practices to cultivating few income generating food crops [26].

**Table 3:** Shannon-Wiener Diversity Index (H’) and Evenness (J) for the seven study sites in Sebeta-Awas District

Study sites	Species richness	Shannon’s index (H’)	Evenness (H’/H’ max)
Dima Guranda	35	3.555	1.000
Dima Magno	29	3.367	0.947
Haro Jilla	33	3.497	0.984
Dalety	18	2.89	0.813
Geja Migira	19	2.994	0.842
Kotche	32	3.446	0.969
Korke	25	3.219	0.905

### Diversity of uses of homegarden species

**Fig. 2:**
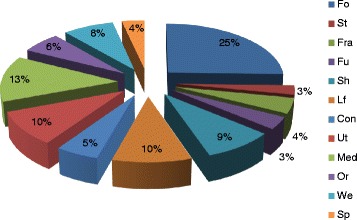
Use category of homegarden plant species in Sebeta-Awas District with their percentage occurrence: Fo = food crop, St = stimulant, Fra = fragrant, Fu = fuel wood, Sh = shade, Lf = Life fence, Con = construction, Ut = household tools, Med = medicine, Om = ornamental, Wd = weed, Sp = spice

It was found that food plants (fruits, vegetables, legumes and pulses) constituted 25 %, of the recorded homegarden species (Fig. 2), which is in agreement with findings of studies conducted in Sabata town of Ethiopia [26] and in Loma and Gena Bosa districts of Ethiopia [8] where the most frequently maintained crops in the homegardens of Sabata town were reported to be those that serve as source of food. In the study area, medicinal and household tool plants comprised 13 and 10 % of the total reported plants, respectively.

#### Food plants

Food plants are cultivated in homegardens year round in the study area although most of these plants are available in adequate amount only during the main rainy season (June and September). Five nutraceutical plants were recorded from homegardens in the study area (Table 4). Paired comparison exercise conducted on all the five nutraceutical plants revealed *Allium sativum* and *Ensete ventricosum* as the most important nutraceutical plants (Table 5). The two species are the ones that are commonly used as nutraceuticals in Ethiopia as revealed in studies conducted elsewhere in the country [26, 40].

**Table 4:** Homegarden nutraceutical plants recorded from the Sebeta-Awas District

Botanical name	Part used	Ailment treated	Method of preparation and use
*Allium sativum*	Bulb	Headache, abdominal cramp and flue	The bulb is eaten alone and/or pounded together with *Zingiber officinale*
*Carica papaya*	Seed	Intestinal parasites	Fresh seeds are eaten
*Cucurbita pepo*	Seed	Tape worm infection	Roasted seeds are chewed and swallowed
*Ensete ventricosum*	Corm	Broken legs and arms	The underground part is boiled and eaten
*Punica granatum*	Leaf	Tape worm infection	Decoction of leaves is drunk

**Table 5:** Results of paired comparisons on five homegarden nutraceutical plants in Sebeta-Awas District

Medicinal plant name	Informants (coded R1 to R12)														Total score	Ranking
	R1	R2	R3	R4	R5	R6	R7	R8	R9	R10	R11	R12	R13	R14		
*Allium sativum*	3	3	3	4	3	2	3	3	3	3	2	2	4	3	41	1
*Carica papaya*	1	2	0	0	1	0	2	3	0	1	1	2	2	3	18	4
*Cucurbita pepo*	2	2	2	2	2	3	3	1	3	3	3	2	0	2	30	3
*Ensete ventricosum*	3	2	4	3	3	4	2	1	3	2	3	3	2	2	37	2
*Punica granatum*	1	1	1	1	1	1	0	2	1	1	1	1	2	0	14	5

#### Medicinal plants

People of Sebeta-Awas District are dependent on medicinal plants grown in their homegardens in to partly fulfill their day-to-day health care needs. Twelve percent of the plants recorded from homegardens are medicinal plants. Of the medicinal plants, herbs were the most common used ones, followed by shrubs. Study conducted in homegardens of the Holeta town of Ethiopia also indicated the common use of herbs as sources of medicine [40].

#### Marketed plants

Homegardens support families in generating additional income although income varied with size of the homegardens. Farmers of Sebeta-Awas District who were in close vicinity to roads and local markets (Sebeta and Alemgana) gave priority to grow cash crops such as *Catha edulis*, *Rhamnus prinoides* and *Rosmarinus officinalis* in their homegardens. The role of home gardens in generating income to families in Ethiopia has also been reported by different authors [8, 26, 40, 41]. Focus to grow few cash crops by neglecting other beneficial crops could reduce the diversity of species managed in homegardens. Simple preference ranking exercise conducted on seven marketed homegarden plants in Sebeta-Awas District with the highest frequencies and relative frequencies of occurrence revealed *Catha edulis* as the most valued marketed homegarden plant, followed by *Rosmarinus officinalis* and *Rhamnus prinoides* (Table 6). The stimulant plant *Catha edulis* has been reported as one of three homegarden plants that generate the highest income in Holeta town of Ethiopia [40].

**Table 6:** Simple Preference ranking exercise on seven marketable homegarden plants in Sebeta-Awas District with the highest frequencies and relative frequencies of occurrence

Botanical name	Respondents (R)															
	R1	R2	R3	R4	R5	R6	R7	R8	R9	R10	R11	R12	R13	R14	Total score	Rank
*Artemisia absinthium*	3	2	1	1	1	2	2	1	2	1	1	1	2	2	22	7
*Catha edulis*	7	6	7	7	7	7	7	7	7	4	6	7	6	7	92	1
*Cymbopogon citratus*	2	1	3	2	2	1	1	2	3	3	2	2	1	1	26	6
*Myrtus communis*	1	3	2	3	3	3	3	3	1	2	3	4	3	3	37	5
*Rhamnus prinoides*	5	4	5	5	5	5	6	5	4	7	5	6	5	5	72	3
*Ruta chalepensis*	4	5	4	4	4	4	5	4	5	5	4	4	4	4	60	4
*Rosmarinus officinalis*	6	7	6	6	6	6	5	6	6	6	7	5	7	6	85	2

Value 7 was assigned to the most valuable plant while 1 to the least valuable plant

### Similarity among homegardens

Sorenson’s Index of Similarity calculated for homegarden plants in the seven selected kebeles revealed that there is highest similarity between homegardens of Kotche and Geja Migira kebeles, followed by that of Dalety and Dima Guranda (Table 7).

**Table 7:** Sorenson’s Index of similarity of homegarden species among seven selected kebeles of Sebeta-Awas District

Study kebele	DG	DM	HJ	DA	GM	KO	KOC
DG	1.00						
DM	0.66	1.00					
HJ	0.60	0.66	1.00				
DA	0.73	0.72	0.66	1.00			
GM	0.58	0.50	0.57	0.66	1.00		
KO	0.70	0.47	0.50	0.50	0.66	1.00	
KOC	0.63	0.66	0.80	0.27	0.81	0.66	1.0

*DG* Dima Guranda, *HJ* Haro Jila, *DM* Dima Magno, *DA* Dalety, *GM* Geja Migira, *KO* Korke, *KOC* Kotche

### Multipurpose tree species

Local people in the study District grow in their homegardens plants having diverse uses. Direct matrix ranking exercise conducted on six multipurpose tree species selected during ethnobotanical survey showed *Eucalyptus globulus* as the most preferred multipurpose tree species, followed by *Juniperus procera* and *Acacia abyssinica* (Table 8). A direct matrix ranking exercise conducted by respondents in Holeta town of Ethiopia revealed *Juniperus procera* as the most preferred multipurpose tree species in homegardens [40].

**Table 8:** Result of direct matrix ranking exercise conducted on six multipurpose homegarden tree species in Sebeta-Awas District

	*Cordia africana*	*Croton macrostachyus*	*Acacia abyssinica*	*Acacia albida*	*Juniperus procera*	*Eucalyptus globulus*
Construction	40	21	32	36	64	74
Soil fertility	38	30	52	60	28	-
Furniture/ Implements	65	42	48	38	42	28
Charcoal/ fire wood	34	26	60	56	25	59
Shade	48	36	50	32	38	24
Live fence	31	22	32	24	66	59
Medicine	35	62	26	18	38	63
Total score	291	229	300	254	301	307
Rank	4	6	3	5	2	1

### Homegarden plants with the highest frequency of occurrence

Marketed homegarden plants were assessed for their frequencies and relative frequencies of occurrence. It was found out that *Catha edulis* had the highest frequency and relative frequency of occurrence, followed by *Rosmarinus officinalis* and *Rhamnus prinoides*. Seven marketed homegarden plants with the highest frequencies and relative frequencies of occurrence are given in Table 9. Result of another study conducted in Jibithenan District, Ethiopia, showed *Catha edulis* as one of the top five most abundant woody species in homegardens [41].

**Table 9:** Lists of seven homegarden plants in Sebeta-Awas District with the highest frequencies and relative frequencies of occurrence

Botanical name	Frequency in percent	Relative frequency in percent
*Artemisia absinthium*	45.2	1.63
*Catha edulis*	80.9	2.94
*Cymbopogon citratus*	52.3	1.89
*Myrtus communis*	47.6	1.73
*Rhamnus prinoides*	73.8	2.68
*Ruta chalepensis*	71.4	2.59
*Rosmarinus officinalis*	78.5	2.85

### Homegarden multipurpose tree species with highest density of occurrence

Among multipurpose tree species in the study area, *Cupressus lusitanica* was found to be the most abundant one with the highest relative density, followed by *Eucalyptus camaldulensis* and *Eucalyptus globulus* (Table 10). The reason for this may be due to the availability of seedlings at local market and nursery sites in the nearby Sabata town. A study [26] revealed *Cupressus lusitanica* as having the highest relative density among homegarden tree species in Sabata town of Ethiopia

**Table 10:** Multipurpose tree species in Sebeta-Awas District having the highest densities and relative densities of occurrence

Botanical name	Abundance/number	Density	Relative density %
*Cupressus lusitania*	250	0.0264	0.0042
*Eucalyptus camaldulensis*	92	0.0097	0.0015
*Acacia albida*	23	0.0024	0.0002
*Eucalyptus globulus*	81	0.0085	0.0013
*Olea europaea* subsp. *cuspidat*a	29	0.0030	0.0004
*Cordia africana*	21	0.0022	0.0003
*Juniperus procera*	28	0.0020	0.0003
*Grevillea robusta*	68	0.0071	0.0011
*Acacia abyssinica*	17	0.0017	0.0002

### Factors affecting species diversity and productivity of homegarden plants

Main factors affecting the diversity and productivity of homegardens plants in Sebeta-Awas District have been reported by respondents. These, among others, include lack of access to water, size of the homegarden, and occurrence of pests and weeds. Shortage of water was mentioned as the main constraint in growing homegarden crops. According to the informants, homegardens in the study area were primarily dependent on rain and as a result diversity and productivity of plants was highly affected during the dry season. Water fetching from distant areas and use of irrigation have been reported to be laborious and time-consuming task. As explained by some informants, the effect of pests on some homegarden plants could not be undermined. *Galium aparinoides* and *Parthenium hysterophorus* were among the weeds that affect the diversity and productivity of homegarden plants. As noted by many authors, there are a number of factors affecting development of productive homegardens. These include socio-cultural and economic factors [2, 42–46], ecological factors and farmers’ knowledge and awareness [47], access to land [48, 49] and labour inputs [7].

## Conclusions

Homegardens are still playing important role in the overall farming system in Sebeta-Awas District. The fact that the majority of households in the District have homegardens shows that gardening is considered important by farmers as it contributes towards ensuring household food security and income generation. The contribution of individual gardens to biodiversity conservation in the study District should also not be underestimated. In addition, homegardens provide important ecological, social, and cultural functions. Species diversity of homegarden plants in Sebeta-Awas District is affected by a number of factors. Water is the main factor limiting species richness and diversity as water is not always sufficiently available. Because of the need for large field crops as well as scarcity of land, there has been over time, decrease in homegarden plot size. People in the study area largely cultivate homegarden plants, which have better market values. Based on findings of the current research, it is recommended that farmers should be encouraged to manage their homegardens as homegardens play important role in ensuring food security and increasing household income. Community members in the study area should, therefore, be made aware of the role of homegarden plants in fulfilling their nutritional and other requirements as many seem to be not very much aware of such fact.
